# Tim-3 promotes tube formation and decreases tight junction formation in vascular endothelial cells

**DOI:** 10.1042/BSR20202130

**Published:** 2020-10-14

**Authors:** Yizi Cong, Xingmiao Wang, Suxia Wang, Guangdong Qiao, Yalun Li, Jianqiao Cao, Wenguo Jiang, Yuxin Cui

**Affiliations:** 1 Department of Breast Surgery, The Affiliated Yantai Yuhuangding Hospital of Qingdao University, 20 Yudong Road, Yantai, Shandong 264001, P.R. China; 2 Department of Pathology, The Affiliated Yantai Yuhuangding Hospital of Qingdao University, 20 Yudong Road, Yantai, Shandong 264001, P.R. China; 3 Cardiff China Medical Research Collaborative, School of Medicine, Cardiff University, Heath Park, Cardiff CF14 4XN, U.K.

**Keywords:** angiogenesis, endothelial cells, tight junction, Tim-3

## Abstract

As a negative immune checkpoint molecule, T-cell immunoglobulin domain and mucin domain containing molecule-3 (Tim-3) has been found to serve a crucial role in immune escape and tumour progression. Previous studies have reported that Tim-3 is important to endothelial cells and it has also been demonstrated to be involved in numerous types of human diseases, including melanoma, lymphoma, rickettsial infection and atherosclerosis; however, its exact mechanism of action remains largely unknown. In the present study, Tim-3 was overexpressed in vascular endothelial human lung microvascular endothelial cells (HMVECs) and human umbilical vein endothelial cells (HUVECs), and *in vitro* assays were used to determine that Tim-3 promoted cell proliferation, migration, invasion and tube formation through activating cyclin D1 (CCND1), Ras homolog gene family member A and vascular endothelial growth factor (VEGF) receptor 2 (VEGFR2). Additionally, Tim-3 decreased tight junction (TJ) formation and the transepithelial resistance (TER) of endothelial cells by decreasing the expression levels of TJ protein 2, Occludin and claudin 1 (CLND1). In conclusion, these findings suggested that Tim-3 may exert a positive role in angiogenesis and a negative role in TJ formation in vascular endothelial cells, which may provide novel strategies for the treatment of Tim-3-associated diseases.

## Introduction

The discovery that T-cell immunoglobulin and mucin family molecules are associated with numerous types of disease, including allergy, autoimmunity [1] and cancer [2], has attracted increasing attention in recent years. In humans, the Tim family consists of T-cell immunoglobulin domain and mucin domain containing molecule (Tim)-1, Tim-3 and Tim-4, which are all located on chromosome 5q33.2 [1]. Tim-3, also known as the hepatitis A virus cellular receptor 2 (HAVCR2), is an important negative immune checkpoint that serves a critical role in regulating immune cell activity [3]; it is found to be expressed on numerous types of immune cells, including activated T helper type 1 (Th1) cells [4], dendritic cells (DCs) and monocytes/macrophages [5,6]. Tim-3 activation has been observed to reduce T cell-mediated cytotoxicity and is perceived as a promising target for cancer immunotherapy [7]. Moreover, Tim-3 is also overexpressed in several types of solid tumours [8,9] and the ectopic expression of Tim-3 in tumour cells has been associated with a more advanced pathological T-cell classification [10], lymph-vascular invasion [11], lung metastasis [12] and lymphatic metastasis [13]. Besides, a meta-analysis study revealed that increased expression levels of Tim-3 in solid tumours predicted a significantly shorter overall survival [2]; thus, Tim-3 is suggested to serve as a prognostic indicator for patients.

Tim-3 is also expressed on endothelial cells [14,15] and has been reported to serve a role in melanoma [15], lymphoma [16], rickettsial infection [17] and atherosclerosis [18,19]. For example, Tim-3-expressing melanoma endothelial cells were found to increase the tumour cell metastatic potential through facilitating cell intravasation and extravasation [15]. Tim-3 expression in lymphoma-derived endothelial cells facilitated the growth and dissemination of lymphoma through interacting with circulating T cells and suppressing the activation of CD4^+^ T cells; clinically, the expression levels of Tim-3 in the endothelium of B-cell lymphoma were also observed to be correlated to dissemination and a poor prognosis [16]; and increased expression levels of Tim-3 facilitated intracellular rickettsial killing in endothelial cells during the early phase of rickettsial infection [17]. In addition, Tim-3 was demonstrated to act as a negative regulator of atherosclerosis, which was accompanied by increased levels of circulating monocytes and lesional macrophages and decreased levels of regulatory T cells and regulatory B cells [18]. Tim-3 also protected human umbilical vein endothelial cells (HUVECs) from ox-low-density lipoprotein (LDL)-induced apoptosis via the c-Jun N-terminal kinase (JNK) pathway and reversed the inhibitory effect over migration [19]. Tim-3 was also revealed to inhibit ox-LDL-induced inflammatory cytokine production; however, this was due to its suppression over NF-κB activation [19]. Overall, these findings suggested that Tim-3 may serve an important role in endothelial-related diseases in humans.

Our previous study revealed that the overexpression of HAVCR-1 (Tim-1) resulted in reduced tight junction (TJ) formation in human endothelial cells by co-localising with zonula occludens proteins including zonula occludens-1 (ZO-1) and zonula occludens-2 (ZO-2) [20]. Therefore, in the present study, the effects of Tim-3 on cell proliferation, migration, invasion and tube formation were investigated in human lung microvascular endothelial cells (HMVECs) and HUVECs, and subsequently, the potential role of Tim-3 in TJs was analysed.

## Materials and methods

### Cell lines and culture

HMVECs (Catalog #: CC-2543) and HUVECs (Catalog #: C2519A) were purchased from Lonza (Slough, U.K.). They were cultured in Dulbecco’s modified Eagle’s medium (DMEM)/Ham’s F-12 with l-Glutamine (Sigma–Aldrich; Merck KGaA), supplemented with 10% FBS (Sigma–Aldrich; Merck KGaA) and antibiotics (Sigma–Aldrich; Merck KGaA). Cells were maintained at 37°C and 5% CO_2_ in a humidified incubator.

### Establishment of stable cell lines overexpressing Tim-3

To establish Tim-3 overexpressing endothelial cells, the full length of Tim-3 (PLV [Exp]-EGFP: T2A: Puro-CMV>hHAVCR2 [NM_032782.4]) or negative control scramble (PLV [Exp]-EGFP: T2A: Puro-CMV>stuffer_300 bp) was transfected into HMVECs and HUVECs using a lentivirus vector (VectorBuilder Inc.). Briefly, 5 × 10^4^ cells were seeded into a six-well plate and medium containing 10 μg/ml polybrene and lentiviral vector was added the following day. The medium was removed and replaced with normal medium following incubation for 20 h. A total of 2 μg/ml puromycin (Sigma–Aldrich; Merck KGaA) was used following culture for 3 days to select the stable cell lines. Following the selection, the cells were cultured in normal medium containing 0.25 μg/ml puromycin.

### Reverse transcription-quantitative PCR

Total RNA was extracted from cultured cells using TRIzol® reagent (Sigma–Aldrich; Merck KGaA) and was reverse transcribed into cDNA using the GoScript™ Reverse Transcription System kit (Promega Corporation) [21], according to the manufacturer’s protocol. qPCR was subsequently performed using an iCycler iQ™ (Bio-Rad Laboratories, Inc.). The following thermocycling conditions were used for the qPCR: 94°C for 5 min and 100 cycles of 94°C for 10 s, 55°C for 35 s and 72°C for 20 s. The following primer sequences used are presented in Table 1. All the primers were synthesised by Sigma–Aldrich (Irvine, U.K.). They were dissolved using dd H_2_O to 100 µM and stored at −20°C. mRNA expression levels were quantified using the 2^−Δ*Ct*^ method and normalised to GAPDH.

**Table 1:** Primer sequences used in the reverse transcription-quantitative PCR

Gene	Forward primers (5′–3′)	Reverse primers (5′–3′)
*GAPDH*	CTGAGTACGTCGTGGAGTC	**ACTGAACCTGACCGTACA**CAGAGATGATGACCCTTTTG
*Tim-3*	GCTCCATGTTTTCACATCTT	**ACTGAACCTGACCGTACA**ATTCCACTTCTGAGGACCTT
*CCND1*	CGGTGTCCTACTTCAAATGT	**ACTGAACCTGACCGTACA**CAAAGCGGTCCAGGTAGTTC
*Rho A*	CTGCTCTGCAAGCTAGACG	**ACTGAACCTGACCGTACA**CAAGACAAGGCAACCAGAT
*ZO-2*	CAAAAGAGGATTTGGAATTG	**ACTGAACCTGACCGTACA**GAGCACATCAGAAATGACAA
*Occludin*	GAATTCAAACCGAATCATTG	**ACTGAACCTGACCGTACA**TGAAGAATTTCATCTTCTGG
*CLDN1*	GAAGTGTATGAAGTGCTTGG	**ACTGAACCTGACCGTACA**CAGACCTGCAAGAAGAAATA

Z sequence ‘ACTGAACCTGACCGTACA’ is highlighted in bold font.

### Western blotting

Total protein was extracted from cells using a protein lysis buffer. Total protein was quantified using a Bio-Rad DC protein assay kit (Bio-Rad Laboratories, Inc.) and equal amounts of protein were separated via SDS/PAGE. The separated proteins were subsequently transferred to a polyvinylidene difluoride (PVDF) membrane and blocked with 5% skimmed milk for 2 h. The membranes were incubated with the following primary antibodies: anti-Tim-3 antibody (cat. no. ab241332; Abcam), anti-GAPDH (cat. no. sc-47724; Santa Cruz Biotechnology, Inc.), anti-cyclin D1 (CCND1) (cat. no. sc-8396; Santa Cruz Biotechnology, Inc.), anti-ZO2 (cat. no. sc-11448; Santa Cruz Biotechnology, Inc.) and anti-Occludin (cat. no. sc-133256; Santa Cruz Biotechnology, Inc.). Following the primary antibody incubation, the membranes were incubated with anti-mouse (cat. no. A5278; Sigma–Aldrich; Merck KGaA), anti-rabbit (cat. no. A0545; Sigma–Aldrich; Merck KGaA) and anti-goat (cat. no. A8919; Sigma–Aldrich; Merck KGaA) secondary antibodies. Protein bands were visualized using Luminata Forte (Merck KGaA) and quantified using ImageJ software (National Institutes of Health) based on the band intensities.

### Cell proliferation assay

Cell proliferation was assessed using the alamarBlue assay. Briefly, 2 × 10^3^ cells/well were seeded into a 96-well plate and incubated for 6 days; the medium was replaced every 3 days during this period. Following incubation for 2, 4 and 6 days, the medium (15 wells per group) was aspirated and 100 μl fresh medium, containing 10 μl alamarBlue (Bio-Rad Laboratories, Inc.) was added to each well and incubated for 3 h at 37°C. The fluorescence was detected using a fluorescence plate reader (Promega Corporation), using an excitation wavelength of 525 nm and an emission wavelength of 590 nm. The percentage growth following the incubation period was then calculated against the overnight plates.

### Wound healing assay

A total of 2 × 10^5^ cells were seeded in a 24-well plate and cells were subsequently scratched using a 1-ml pipette tip to generate an artificial wound in the cell monolayer. After washing the cells twice with PBS, normal medium was added to each well. The migratory ability was monitored using an EVOS® FL imaging system (Thermo Fisher Scientific, Inc.) at a 4× objective every 2 h for 24 h. The percentage wound closure at the experimental end point compared with at 0 h was analysed using ImageJ software.

### Matrigel invasion assay

The cell invasive ability was assessed using an *in vitro* Matrigel invasion assay. Briefly, Transwell inserts (8-μm pores) for 24-well plates were precoated with 100 μl/insert of 0.5 mg/ml Matrigel (BD Biosciences) for 1 h at 37°C. Subsequently, a total of 2 × 10^4^ cells were plated in the upper chambers of Transwell plates in 150 μl DMEM. A total of 650 μl normal medium was plated in the lower chambers. Following incubation for 48 h, non-invasive cells remaining in the upper chambers were removed with a cotton swab. The invasive cells in the lower chambers were fixed with 4% formalin for 30 min and stained with 1% Crystal Violet for 30 min, before rinsing with phosphate-buffered saline (PBS). Stained cells were counted under a microscope with more than or equal to five counts per experimental setting.

### Cell-matrix adhesion assay

A 96-well plate containing Matrigel (10 μg/well) was incubated at 37°C for 2 h. A total of 2 × 10^4^ cells/well were added and incubated for 1 h and then washed twice with PBS. Adhesive cells were fixed with 4% formalin and stained with 1% Crystal Violet before rinsing with PBS. The number of attached cells was counted under a microscope with more than or equal to five counts per experimental setting.

### Tube formation assay

Prechilled 96-well plates were coated with 50 µl/well Matrigel (BD Biosciences) and incubated to polymerise at 37°C for 1 h. A total of 2 × 10^4^ cells were plated into each well and incubated at 37°C and 5% CO_2_ for 16 h. Five views from five wells of each group were then captured to evaluate the tube formation ability by counting the total segments length automatically using ImageJ software. A segment was defined as an element delimited by two junctions of the newly formed tubule network.

### Electric cell-substrate impedance sensing assay

The electric cell-substrate impedance sensing (ECIS) Zθ system with 96W1E+ array plate (Applied BioPhysics, Inc.) was used to measure the initial attachment and spreading of cells. Briefly, the plate was stabilised using normal medium for 2 h and 5 × 10^4^ cells/well were seeded and cultured for 24 h. The resistance across the array was recorded at different frequencies.

### Transepithelial resistance and paracellular permeability assays

An EVOM Voltohmmeter (World Precision Instruments), equipped with STX2 chopstick electrodes (World Precision Instruments) was used to measure the transepithelial resistance (TER). Briefly, 5 × 10^4^ cells were plated into a 0.4-μm pore size insert (Greiner Bio-One Ltd) and cultured to 100% confluence. Electrodes were placed in the upper and lower chambers and resistance was subsequently measured using a Volt-Ohm meter. Inserts without cells in medium were set as a blank control. Following the analysis of TER, the medium in the upper chambers was replaced with normal medium containing 0.2 mg/ml fluorescein isothiocyanate (FITC)-dextran 10 kDa. Then, 50 µl medium from outside the insert was transferred into a black 96-well cell culture microplate (Greiner Bio-One) in duplicate every 2 h for 10 h. The basolateral dextran passage was analysed using a GloMax®-Multi Microplate Multimode reader (Promega Corporation), with an excitation wavelength of 490 nm and an emission wavelength of 510–570 nm. Each measurement was normalized to the 0 h via subtraction.

### Enzyme-linked immunosorbent assay

Cultured cells were concentrated using the Amicon Ultra-4 centrifugal filter units (Sigma–Aldrich; Merck KGaA) and the medium was subsequently used for enzyme-linked immunosorbent assay (ELISA). ELISA was performed using the human vascular endothelial growth factor (VEGF) receptor 2 (VEGFR-2) ELISA kit (cat. no. E-EL-H1603; Elabscience), according to the manufacturer’s protocol.

### Bioinformatics analysis

The transcription level correlation of Tim-3 with angiogenic regulators was investigated using a pooled analysis of publicly available endothelial (single) cell gene expression data from the EndoDB version 05/07/2019 (https://endotheliomics.shinyapps.io/endodb/). Only studies on primary or freshly isolated endothelial cells were selected, and untreated or control groups were recruited, which provided a cohort with 643 samples. The correlation coefficients (R) were measured using Pearson’s correlation method.

### Immunohistochemistry assay

Breast cancer tissues were collected after surgical resection at the Affiliated Yantai Yuhuangding Hospital of Qingdao University with written consent forms from each patient. The protocol was approved by the local Research Ethics Committee. The immunohistochemistry (IHC) assay was performed following our standard operation protocol using a Tim-3 antibody (ab241332. Abcam, Cambridge, U.K.).

### Statistical analysis

Statistical analysis was performed using GraphPad Prism version 7.0 software (GraphPad Software, Inc.) and data are presented as the mean ± SD. For the ECIS data with repeated measures (RMs) of time-lapse, statistical differences between two groups were analysed using two-way ANOVA with RM and multiple comparisons. The post-hoc test following the RMs ANOVA was Tukey’s multiple comparisons test. For other experimental data, group comparison was performed using a two-sided Student’s *t* test when the data were normally distributed or Mann–Whitney U test when the data were not normally distributed. Experiments were repeated two- to four-times unless otherwise stated. The number of samples per group in each experiment was at least three (*n*=3) if not indicated. *P*<0.05 was considered to indicate a statistically significant difference. *P<0.05; **P<0.01; ***P<0.001.

## Results

### Stable endothelial cell lines overexpressing Tim-3

**Figure 1:**
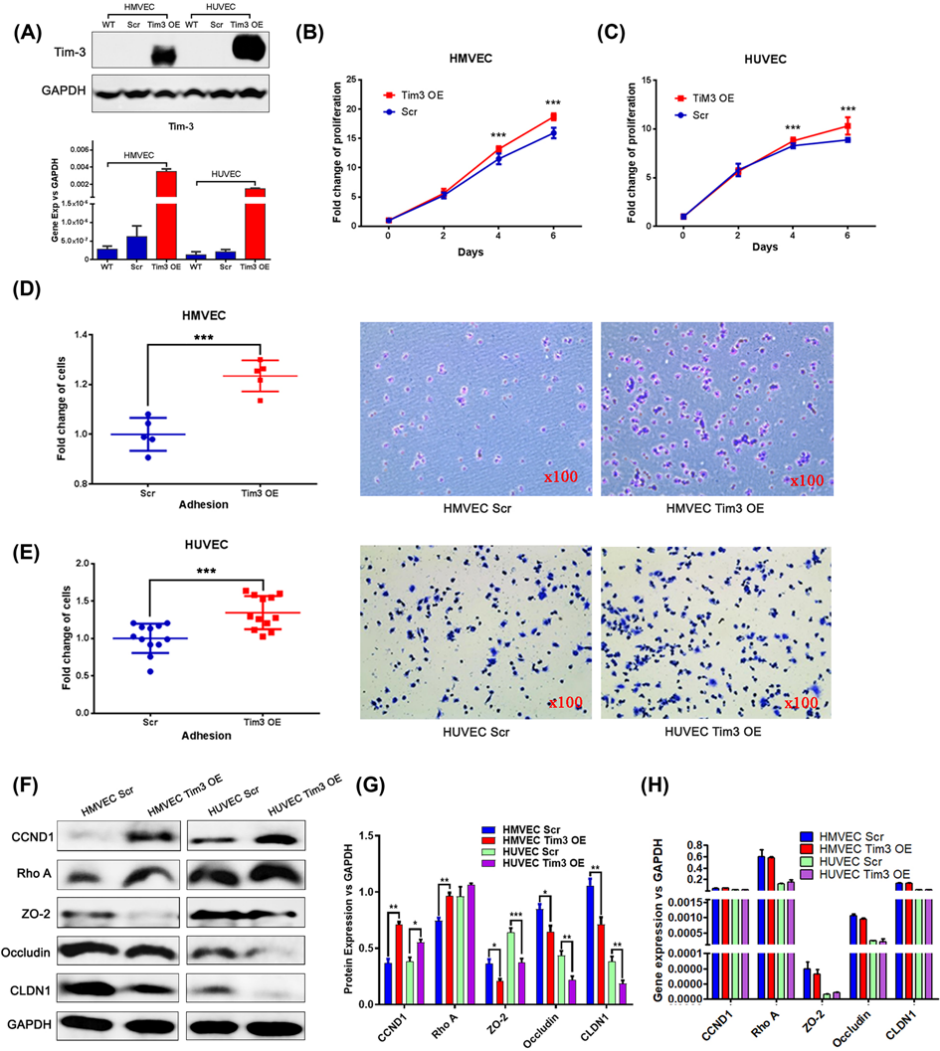
Stable HMVECs and HUVECs overexpressing Tim-3 were established, which were found to increase cell proliferation and adhesion *in vitro* (**A**) Successful overexpression of Tim-3 in HMVECs and HUVECs was analysed using RT-qPCR and Western blotting. (**B,C**) Overexpression of Tim-3 increases cell proliferation in (B) HMVECs and (C) HUVECs. (**D,E**) Overexpression of Tim-3 enhances the cell adhesive ability of (D) HMVECs (*P*<0.001) and (E) HUVECs (*P*<0.001). (**F**) Protein expression levels of CCND1, RhoA, ZO-2, Occludin and CLDN1 in Scr and Tim-3 OE cells were analysed using Western blotting. Following normalisation of the cell lysate samples, the Western blots were derived from different membranes with equal-amount sample loading and GAPDH blots as a control. (**G**) Semi-quantitative analysis of Western blotting using ImageJ software. (**H**) Gene expression levels of CCND1, RhoA, ZO-2, Occludin and CLDN1 were determined using RT-qPCR. *P<0.05; **P<0.01; ***P<0.001.

HMVECs and HUVECs were stably transfected with full-length Tim-3 overexpression (Tim-3 OE) plasmid or empty vector [Scramble (Scr)] and the expression levels of Tim-3 in the two cell lines were analysed using reverse transcription-quantitative PCR (RT-qPCR) and Western blotting. Tim-3 expression levels were significantly increased in Tim-3 OE cells compared with the Scr and wildtype cells at both the mRNA and protein levels (Figure 1A), which confirmed that the Tim-3 OE plasmid was successfully transfected to the cells.

### Tim-3 promotes cell proliferation and adhesion *in vitro*

The effect of Tim-3 expression on cell proliferation and adhesion *in vitro* was subsequently investigated. Cell proliferation was significantly increased in both Tim-3 OE HMVECs and HUVECs compared with the Scr cells when cultured for 4 or 6 days (all *P*<0.001; Figure 1B,C). The adhesive ability of cells was assessed using a cell–matrix adhesion assay; it was observed that the adhesive ability was also increased when Tim-3 expression levels were overexpressed in both cell lines (all *P*<0.001; Figure 1D,E).

CCND1 is an important molecule in cell proliferation, thus it was subsequently investigated whether CCND1 was regulated when Tim-3 was overexpressed. CCND1 expression levels were increased in both Tim-3 OE HMVECs and HUVECs at the protein level (Figure 1F,G), which may partially explain the stimulatory role of Tim-3 in cell proliferation.

### Tim-3 promotes cell migration and invasion *in vitro*

**Figure 2:**
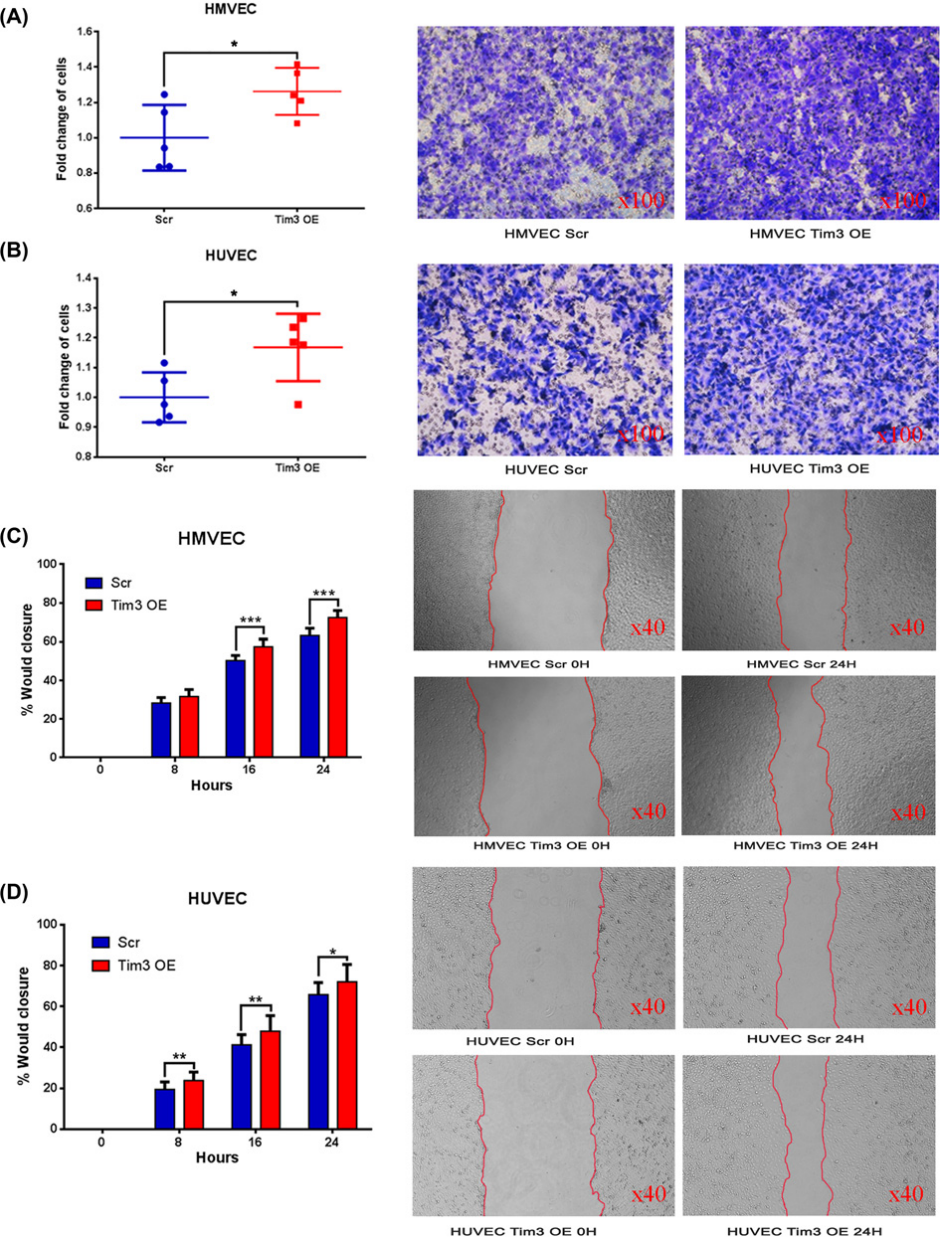
Overexpression of Tim-3 increases cell invasion and migration *in vitro* (**A,B**) Overexpression of Tim-3 increases the invasive ability of (A) HMVECs (*P*=0.033) and (B) HUVECs (*P*=0.030). (**C,D**) Wound healing assay was used to investigate the cell migration ability, which was found to be increased in Tim-3 OE (C) HMVECs (24 h; *P*<0.001) and (D) HUVECs (24 h; *P*=0.026). *P<0.05; **P<0.01; ***P<0.001.

A Matrigel invasion assay and wound healing assay were used to characterise the effects of Tim-3 on cell invasion and migration. The Matrigel invasion assay revealed that Tim-3 OE cells were more invasive compared with Scr cells in both HMVECs (*P*=0.033; Figure 2A) and HUVECs (*P*=0.030; Figure 2B). Similarly, the wound healing assay demonstrated that the cell migration ability was also increased in Tim-3 OE HMVECs at 24 h (*P*<0.001; Figure 2C) and HUVECs at 24 h (*P*=0.026; Figure 2D).

Ras homologue family member A (RhoA) is a critical molecule in the regulation of cell migration, thus it was subsequently investigated whether RhoA was involved in the promotion of cell migration. RhoA was significantly increased when Tim-3 expression levels were overexpressed in both HMVECs and HUVECs (Figure 1F,G).

### Tim-3 promotes tube formation *in vitro*

**Figure 3:**
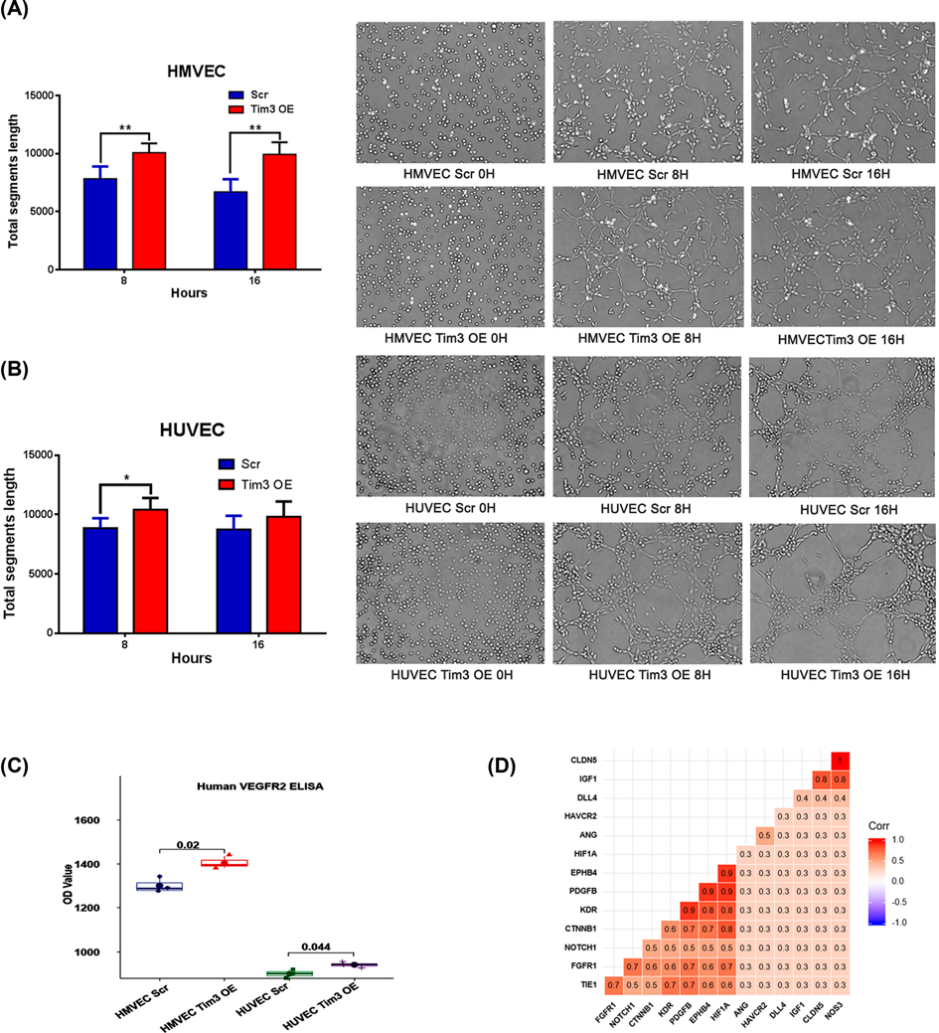
Overexpression of Tim-3 promotes tube formation in vascular endothelial cells (**A,B**) Tube formation ability is increased in Tim-3 OE (A) HMVECs (8 h; *P*=0.005; 16 h; *P*=0.001) and (B) HUVECs (8 h; *P*=0.015). (**C**) VEGFR2 expression levels were analysed using ELISA. (**D**) Correlation analysis between Tim-3 transcriptional levels and angiogenic factors. Endothelial (single) cell gene expression data were extracted from the EndoDB version 05/07/2019 (https://endotheliomics.shinyapps.io/endodb).

The formation of capillary-like tubes by endothelial cells on a membrane matrix is a powerful method to evaluate the capacity of angiogenesis. The tube formation ability was significantly increased in Tim-3 OE HMVECs compared with Scr cells when cultured for 8 h (*P*=0.005) and 16 h (*P*=0.001; Figure 3A). A similar phenomenon was also observed in Tim-3 OE HUVECs when cultured for 8 h (*P*=0.015; Figure 3B), which suggested that the overexpression of Tim-3 may have a potential promoting role over angiogenesis in endothelial cells.

To investigate the mechanisms by which Tim-3 promotes tube formation, a VEGFR2 ELISA was performed using the Tim-3 OE cells and their respective Scr controls. The stable expression of Tim-3 in both HMVECs and HUVECs resulted in increased expression levels of VEGFR2 protein (*P*<0.05; Figure 3C). Besides, bioinformatics analysis using EndoDB revealed that Tim-3 was significantly correlated with the transcription of Angiogenin (ANG), an angiogenic protein (Figure 3D).

### Tim-3 reduces the integrity of endothelial TJs

**Figure 4:**
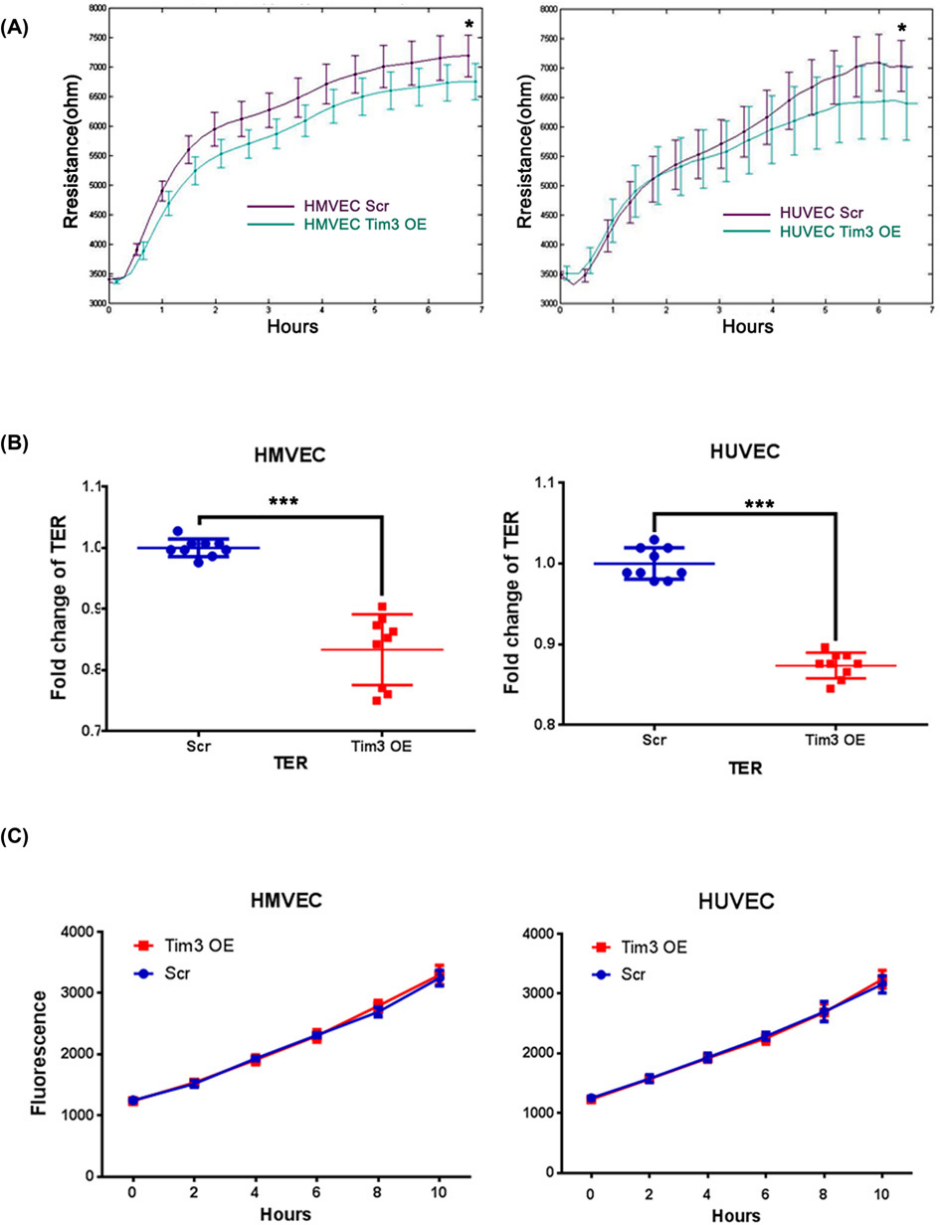
Overexpression of Tim-3 decreases cell–cell TJs in vascular endothelial cells (**A**) Overexpression of Tim-3 promotes a slower initial attachment and spreading of HMVECs and HUVECs, which was assessed using ECIS. (**B**) Overexpression of Tim-3 decreases the TER in both HMVECs (*P*<0.001) and HUVECs (*P*<0.001). (**C**) Overexpression of Tim-3 did not affect the permeability (FITC-dextran 10 kDa) between cells monolayers in HMVECs and HUVECs. *P<0.05; **P<0.01; ***P<0.001.

TJs serve a crucial role in the regulation of endothelial barrier functions and their disruption usually leads to increased paracellular permeability (PCP). Thus, the role of Tim-3 in TJs was investigated. ECIS was firstly used to investigate the initial cell attachment and spreading by monitoring the resistance at 1 kHz; a lower resistance was observed in Tim-3 OE cells compared with Scr cells during the initial attachment and spreading in both HMVECs and HUVECs (Figure 4A), which indicated that Tim-3 may decrease the integrity of cell–cell TJs.

To further confirm our primary findings, TER and PCP assays were performed to investigate the effect of Tim-3 on the TJ barrier function. The TER in Tim-3 OE cells was decreased compared with the control cells in both HMVECs and HUVECs (all *P*<0.001; Figure 4B), which suggested that Tim-3 OE may reduce the cell-to-cell connectivity. Following determination of the TER, PCP FITC-dextran 10 kDa was used to evaluate the permeability between cells monolayers; however, no significant difference was observed between Tim-3 OE cells and Scr cells in both cell lines.

Based on the above findings, the mechanism of Tim-3 in TJs was investigated. From our results, ZO-2, occludin and claudin 1 (CLDN1) expression levels were all discovered to be decreased in both Tim-3 OE HMVECs and HUVECs, as determined using Western blotting (Figure 1F,G), which may partially explain the negative role of Tim-3 in TJs.

### Tim-3 is highly expressed by vascular endothelial cells in breast cancer tissue

**Figure 5:**
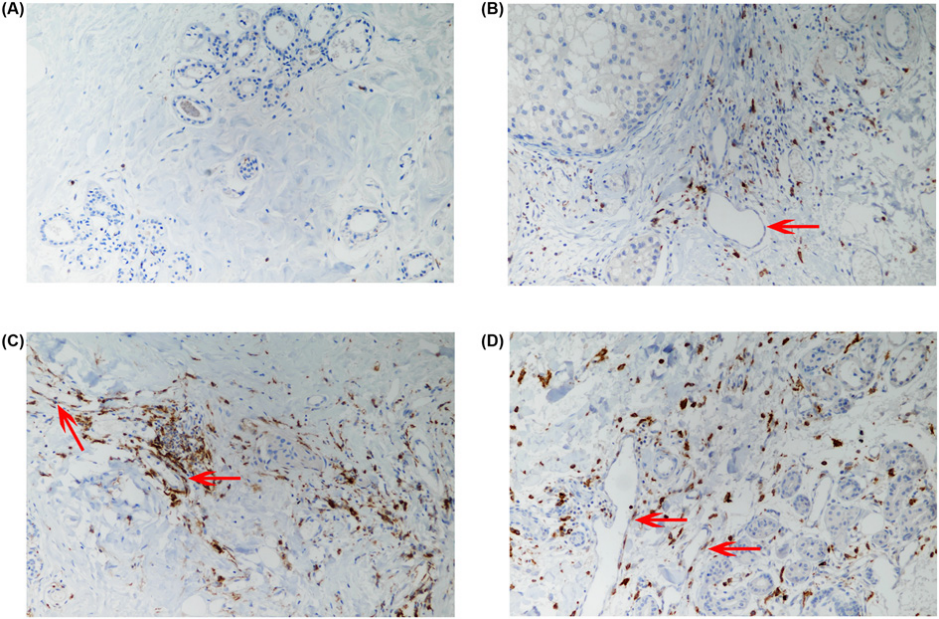
*In vivo* expression of Tim-3 protein in breast cancer tissue indicated by an IHC assay (**A**) No primary antibody control. (**B**) Reprehensive vascular endothelium that is negative for Tim-3. (**C,D**) Reprehensive vascular endothelia that are positive for Tim-3. The representative vascular endothelium sites were highlighted using red arrows.

The cultured human endothelial cells may have a different microenvironment to mediate the expression of Tim-3. We hypothesized that the Tim-3 expression could be altered under certain pathological conditions such as cancer. To examine this we evaluated the expression levels of Tim-3 protein in breast cancer tissue sections by IHC. As shown in Figure 5, Tim-3 was expressed by the vascular endothelial cells in the breast cancer tumour sections with high frequency. This confirmed that there is protein expression of Tim-3 in endothelium *in vivo* at least when malignancy occurred.

## Discussion

Endothelial cells form a one-cell thick monolayer known as the endothelium, which lines the interior surface of blood vessels. Previous studies have demonstrated that the expression of Tim-3 on endothelial cells was associated with several vascular-associated diseases. Therefore, in the present study, Tim-3 was overexpressed in vascular endothelial cells, and *in vitro* functional assays were used to investigate the potential roles and mechanisms of Tim-3.

One of the main functions of the endothelium is to form new blood vessels through a process known as angiogenesis. Angiogenesis has important applications in cancer research, because tumour growth is supported by the creation of new blood vessels. Therefore, current research is focussed on inhibiting the process of angiogenesis to prevent tumour progression. During angiogenesis, dynamic changes in the adhesive structures and mobility are crucial for cellular remodelling. In the present study, the growth of Tim-3 overexpressing cells was significantly enhanced in both HMVECs and HUVECs through activating CCND1. CCND1 promotes cell growth through forming active complexes with either cyclin-dependent kinase 4 (CDK4) or CDK6, which in turn phosphorylates the retinoblastoma protein (Rb) and drives the progression from the G_1_ to S phase [22]. Tim-3 has also been observed to promote cell proliferation through activating the NF-κB signalling pathway in B16 melanoma endothelial cells [15]. The migratory and invasive abilities of cells overexpressing Tim-3 was subsequently investigated and it was discovered that Tim-3 OE could increase the cell migratory and invasive ability in both HMVECs and HUVECs. In B16 melanoma cells, endothelial cell-expressed Tim-3 was reported to increase the cell metastatic potential through facilitating tumour cell intravasation, survival in blood stream and extravasation [15], which supported our results. Cell migration is a multistep process, which serves an important role in numerous types of diseases. An important regulatory element of cell migration is the interdependent regulation of Ras-related C3 botulinum toxin substrate 1 (Rac1) and Rho; Rac1 is required at the front of the cell to regulate actin polymerization and membrane protrusion, whereas Rho appears to regulate the contraction and retraction forces required in the cell body and at the rear [23]. Thus, the current study explored whether RhoA was involved in Tim-3-induced cell migration. Unsurprisingly, RhoA was increased in Tim-3 overexpressing HMVECs and HUVECs, which may partially explain its role in promoting cell migration.

Since Tim-3 was found to exert a promotive role in cell growth, migration and invasion, which are crucial mechanisms involved in angiogenesis, it was subsequently determined whether Tim-3 could promote tube formation *in vitro*. As illustrated from our results, the up-regulation of Tim-3 also increased the tube-forming ability, which suggested that Tim-3 may serve a positive role in angiogenesis. The underlying mechanism by which Tim-3 promotes tube formation may be due to its ability to induce VEGFR production, which therefore enhances the regulation of VEGF; however, Tim-3 may also mediate tube formation in a VEGF/VEGFR independent manner, such as through the ANG signalling pathway.

In a recent study, another co-stimulatory molecule, B7 homologue 3 protein (B7-H3), was also demonstrated to increase cell proliferation, migration and tube formation in HUVECs through promoting VEGF secretion [24]. The vascular endothelium in the tumour microenvironment is essential in tumour progression, thus the fact that Tim-3 can promote angiogenesis may suggest its significance in tumour progression.

Another significant function of the endothelium is to maintain homoeostasis through regulating the permeability of vessels. This property is well researched in relation to the blood–brain barrier system due to the difficulty in developing drugs that can cross the endothelial barrier efficiently. Current research is focussed on better understanding the functions of the blood–brain barrier system. A previous study reported that HAVCR-1 (Tim-1) could down-regulate the apparatus required for TJ formation in human endothelial cells by co-localising with ZO-1 and ZO-2 proteins, which reduced TJ formation [20]. Therefore, TJ function was also investigated in the present study to determine whether Tim-3 was also involved in TJs. ECIS was firstly used to investigate the initial attachment and spreading; a lower resistance was observed in Tim-3 OE cells compared with Scr cells in both HMVECs and HUVECs, which implied that Tim-3 may decrease the integrity of TJs. Subsequently, the TER assay further validated these findings, as a decreased TER was found in Tim-3 OE cells compared with Scr cells in both cell lines; however, no significant difference was found in the monolayer cell permeability between the Scr and Tim-3 OE cells, which was determined using the PCP assay with FITC-dextran 10 kDa. It was suggested that this may be due to the molecular weight (10 kDa) used in our detection.

Important TJ molecules were subsequently analysed to determine the mechanism of Tim-3 in TJs. From our results, ZO-2, Occludin and CLDN1 expression levels were all decreased in both Tim-3 OE HMVECs and HUVECs. ZO-2 is a peripheral TJ protein belonging to the membrane-associated guanylate kinase protein family. Occludin has been reported to directly bind to N-ZO-2, as well as the NH_2_-terminal discs-large-like portion of ZO-1 (N-ZO-1) *in vitro* [25]. Previously, in ZO-1 KO/ZO-2 KD epithelial cells, no TJs were found, although cells retained a polarized distribution of membrane proteins [26]. Therefore, it was hypothesised that Tim-3 may decrease TJ functions through down-regulating ZO-2 expression, which would further down-regulate Occludin and CLDN1 expression levels; however, the mechanism by which Tim-3 down-regulates TJs requires further research in the future.

Migration of vascular endothelial cells is critical in maintaining the integrity of blood vessel walls and leads to the formation of new blood vessels, thereby improving the prognosis of atherosclerosis [27]. Inhibition of vascular endothelial cell migration promotes atherosclerosis progression through suppressing the repair of damaged vascular endothelial cells and angiogenesis. Tim-3 promotes cell proliferation and migration in vascular endothelial cells, suggesting its potential in the treatment of atherosclerosis [19]. We demonstrate that there is an endogenous expression of Tim-3 protein in the vascular endothelial cells from breast cancer tissue. Tim-3 might be up-regulated on vascular endothelium in patients with tumour malignancies due to the stimulation by growth factors in the tumour microenvironment. Tim-3 could promote tube formation and down-regulate TJs *in vitro*, indicating that Tim-3 may play a great role in cancer invasion and metastasis through inducing angiogenesis and increasing capillary permeability. Further investigation will be encouraged though to obtain a deeper insight in the future.

As one of the limitations of the present study, it may be worthy to be noted that Tim-3 knockdown *in vitro* models was not established to check whether Tim-3 knockdown has the opposite function. Future work will be encouraged to estimate the role of Tim-3 in angiogenesis in particular in pathological conditions using an appropriate animal model.

In conclusion, the proliferative, adhesive, migratory and invasive ability were all increased when Tim-3 expression levels were overexpressed in HMVECs and HUVECs through the activation of CCND1 and RhoA. Tim-3 also served a positive role in promoting tube formation and reducing cell–cell TJs and TER through decreasing ZO-2, Occludin and CLDN1 expression levels.

## Data Availability

All data relevant to the present study are given with the main paper, including figures and tables. The primary data that support the findings of the present study are available from the corresponding authors upon reasonable request.

## Abbreviations

ANG: angiogenin
CCND1: cyclin D1
CDK4: cyclin-dependent kinase 4
CDK6: cyclin-dependent kinase 6
CLND1: Claudin 1
DC: dendritic cell
DMEM: Dulbecco’s modified Eagle’s medium
ECIS: electric cell-substrate impedance sensing
ELISA: enzyme-linked immunosorbent assay
FITC: fluorescein isothiocyanate
HAVCR2: hepatitis A virus cellular receptor 2
HMVEC: human lung microvascular endothelial cell
HUVEC: human umbilical vein endothelial cell
IHC: immunohistochemistry
KD: Knock down
KO: Knock out
LDL: low-density lipoprotein
PBS: phosphate-buffered saline
PCP: paracellular permeability
Rac1: Ras-related C3 botulinum toxin substrate 1
RhoA: Ras homologue family member A
RM: repeated measure
RT-qPCR: reverse transcription-quantitative PCR
Scr: Scramble
TER: transepithelial resistance
Tim-3: T-cell immunoglobulin domain and mucin domain containing molecule-3
Tim-3 OE: Tim-3 overexpression
TJ: tight junction
VEGF: vascular endothelial growth factor
VEGFR2: VEGF receptor 2
ZO-1: zonula occludens-1
ZO-2: zonula occludens-2
